# Extracellular vescicles in psoriasis: from pathogenesis to possible roles in therapy

**DOI:** 10.3389/fimmu.2024.1360618

**Published:** 2024-05-17

**Authors:** Marco Iuliano, Lorenzo Grimaldi, Paolo Rosa, Sofia Scibetta, Nicoletta Bernardini, Ilaria Proietti, Ersilia Tolino, Nevena Skroza, Concetta Potenza, Giorgio Mangino, Giovanna Romeo

**Affiliations:** ^1^ Department of Medico-Surgical Sciences and Biotechnologies, Sapienza University of Rome – Polo Pontino, Latina, Italy; ^2^ ICOT, Istituto Chirurgico Ortopedico Traumatologico, Latina, Italy; ^3^ Dermatology Unit “Daniele Innocenzi”, “A. Fiorini” Hospital, Terracina, Italy

**Keywords:** psoriasis pathogenesis, extracellular vesicles, exosomes, inflammatory microenvironment, microRNA

## Abstract

Psoriasis is a chronic inflammatory disease affecting skin and joints characterized by a chronically altered immune and inflammatory response. Several factors occur from the onset to the development of this disease due to different types of cells spatially and temporally localized in the affected area, such as, keratinocytes, macrophages, neutrophils and T helper lymphocytes. This scenario leads to the chronic release of high levels of inflammatory mediators (*i.e.*, IL-17, IL-23, IL-22, TNF-α, S100 proteins, Defensins) and lastly parakeratosis and thickening of the stratum spinosum. Extracellular vesicles (EVs) are small double membraned biological nanoparticles that are secreted by all cell types and classified, based on dimension and biogenesis, into exosomes, microvesicles and apoptotic bodies. Their role as vessels for long range molecular signals renders them key elements in the pathogenesis of psoriasis, as well as innovative platforms for potential biomarker discovery and delivery of fine-tuned anti-inflammatory therapies. In this review, the role of EVs in the pathogenesis of psoriasis and the modulation of cellular microenvironment has been summarized. The biotechnological implementation of EVs for therapy and research for new biomarkers has been also discussed.

## 1 Introduction

Psoriasis is a chronic inflammatory-mediated disease that affects the skin and has an incidence of 2–3% of the world’s population (1). Despite the direct cause behind psoriasis development is still unknown, many risk factors and molecular components have been described. In the beginning, blood vessels enlarge and become tortuous. In this stage the dermis remains unaltered. Soon after, keratinocyte (KC) hyperproliferation can be observed, accompanied by parakeratosis (*i.e*., retention of nuclei by corneocytes). Lastly, during the late stages, acanthosis becomes prevalent. This phenomenon is a result of the KCs invading the higher layers of the skin and the thickening of the *stratum spinosum*, producing a darker tone and a scaly texture (2). A molecular trigger for psoriasis has also been identified. In response to a physical trauma of the skin, KCs release the cationic antimicrobial peptide (AMP) LL-37. DNA or RNA fragments released from the lesion are bound to LL-37 and form LL-37/self-DNA/RNA complexes found in psoriasis lesions, which in turn activate TLR7/9-bearing dendritic cells (DCs). This results in type I interferon (IFN) production and initiation of inflammation. One of the main features of psoriasis is the dysregulated and chronic immune response, stemming from a vicious cycle between damaged KCs, DCs and local T cells. Myeloid DCs in the skin are activated by pro-inflammatory cytokines produced by damaged KCs such as Tumor Necrosis Factor-α (TNF-α) Interleukin- (IL)-1β and IL-6 as well as the LL-37/self-DNA/RNA complex. DCs, then, activate γδ-T cells polarization through the release of TNF-α, IL-23, IL-1β and IL-6. The cycle is closed by matured γδ-T cells who induce keratinocyte aberrant differentiation program through IL-17 and the recruitment of neutrophils and other immune cells. Plaque formation and maintenance is enabled by the DC-macrophage IL-23/IL-17 axis which promotes type 17 T helper (Th) and cytotoxic T lymphocyte effector polarization (3). The causes of psoriasis development can be both genetic and environmental (4). It is important to note that, rather than being direct causes, they represent risk factors for psoriasis and likely play roles with different weights in disease emergence. It is, therefore, clear that psoriasis is a disease that occurs when there is an interplay between environment, genetic predisposition factors, an overactive immune system and altered cell-to-cell intercommunication (2, 5).

Extracellular vesicles (EVs) are a class of small double membraned nanoparticles that are secreted by all cell types and mainly act as vessels for delivering cargo and molecular signals (6). Based on their biogenesis and/or size, EVs can be categorized in three subsets: exosomes or small EVs (s-EVs, 30–150 nm in diameter, originating from the endosomal complex); microvesicles or large EVs (l-EVs 100–1000 nm in diameter, secreted from the cell membrane); apoptotic bodies (APs 50–5000 nm in diameter, originated from plasma membrane blebbing during apoptosis) (6–8). Nucleic acids (*i.e.*, several type of RNAs) and proteins are carried by EVs from a donor to an acceptor cell where modulation of expression is carried out. The range of EV signaling can be restricted to adjacent cells localized in the same tissue as the donor cell but can also be extended to a systemic level. While long range cell-to-cell communication is also possible with cell-free RNAs and proteins, the protection provided by EVs from RNAses and proteases represents a major advantage in long distance signaling (8–10). EVs play a crucial role in the regulation of many patho-physiological processes and the definition of the extracellular environment. During disease development EVs are employed both by the host and the pathogen or cancerous cells: the former to regulate the immune response and induce the polarization of macrophages, neutrophils and of other effector cells; the latter to prime the environment for further colonization. Regarding a typical chronic inflammatory disease, as psoriasis, EVs have roles in the regulation of the immune response but also as platforms for therapeutic applications and delivery of innovative drugs. Furthermore, the use of EVs has also had ramifications in disease diagnosis. In recent times, RNA and proteins carried by EVs through the blood stream have been profiled to find molecular biomarkers positively correlated with many diseases. Indeed, such biomarkers have become useful in keeping track of disease progression and amelioration (11–14). The latter aspect is even more important in the case of psoriasis where the patient evaluation and follow-up is still based on clinical criteria, mainly the Psoriasis Area and Severity Index (PASI) and the Dermatology Life Quality Index (DLQI). Hence, the push to molecular characterization of EVs in subjects affected by psoriasis to identify markers for patient management, possibly through a liquid biopsy approach.

Here a selection of the studies about the influence and the potential role of EVs both in the development of psoriasis and in therapeutic intervention to contrast this disease, has been presented. In particular, the review highlights the role of EVs in the composition of psoriasis-associated secretome and microenvironment also suggesting the EV involvement in the spreading of the disease mediators and in the development of associated comorbidities.

## 2 EV release and composition

While it is not clear if EV production is influenced by psoriasis and by its severity (15), *in vitro* experiments with HaCaT cells performed by Mangino et al. have reported that IL-17A treatment significantly alters the rate of EVs production (16). Moreover, the size of the produced EVs was also subjected to modulation since an enrichment in EVs below 450 nm in diameter was observed. Besides IL-17A, other cytokines have been proven to have an effect in EV release, both in size and cargo. To this end, Capriotti et al. (17) carried out experiments by stimulating HaCaT KCs with recombinant chemokines (*i.e*., IFN-γ, TNF-α, IL-17A, IL-22 and IL-23). EVs were subsequently isolated and analyzed through Nanoparticle Tracking Analysis (NTA). The results revealed that only IL-17A and INF-γ modulated EV production, increasing the quantity of EVs below 200 nm produced. Recently, EVs have proven to be valuable elements to keep track of disease progression and development. In this case psoriasis is not an exception and there are many examples in which EVs have been used to monitor both amelioration after treatment and disease development. In fact, it has been proposed that the concentration of EVs in psoriatic patients’ sera is indicative of the chronicity of the disease (18–20).

The molecular cargo of the EVs is the result of an active process orchestrated by specific pathways for exosomes and microvesicles (21). The evaluation of the EVs content can be useful for the discovery of new biomarkers and for determining the possible role of EVs’ cargo in transferring competencies to the acceptor cell.

A study based on proteomic analysis on exosome from KCs stimulated by psoriasis related inflammatory cytokines demonstrated that 72 (10.9%) proteins were up-regulated and 96 (14.5%) down-regulated. The Gene Ontology (GO) analysis showed that the enrichment was focused on proteins linked to the immune effector process, inflammatory response, endocytosis and in molecules involved in serine hydrolase activity, serine-type peptidase activity and heparin binding. The Kyoto Encyclopedia of Genes and Genomes (KEGG) pathways analysis highlighted an increase of proteins related to Wingless-related integration site (Wnt), Nuclear Factor kappa B (NF-κB), cytokine-cytokine receptor and Toll Like Receptor (TLR) pathways (22). The microenvironmental stress can lead to a change in exosome composition even if the abundance of Heat Shock Proteins (HSPs) and S100 proteins remains univariate and there is not a specific molecular inducer of neutrophils activation. This scenario reinforces the hypothesis that the exosomes are subjected to different methods of spreading messages from the producer cells (22). One of the most important mediators of the psoriatic disease is the IL-17, a cytokine produced by Th17 lymphocytes and KCs. The composition of psoriasis-derived exosomes is dependent on the severity of the disease. It has been discovered that IL-17A expression increases 5 times from mild to severe psoriasis (15). The altered regulation of the inflammation can be due to different mechanisms. The control of the oxidative status is fundamental to reduce the increase of reactive oxygen species (ROS) that can trigger the induction of inflammation (23). It has been observed that while the levels of iron and TIBC (Total iron-binding capacity) were significantly lower in psoriasis patients compared to healthy donors, Soluble transferrin receptor (sTfR) and Heme Oxygenase-1 (HO-1) were over-expressed. HO-1 is an inducible enzyme which converts heme into iron, carbon monoxide and biliverdin/bilirubin (24). HO-1 plays fundamental roles in cytoprotection, membrane cholesterol and oxysterols metabolism (25), and in the development of the psoriatic lesion by negatively regulating Stat3 signaling (26). The function of HO-1 is mainly protective against oxidative stress and inflammation (23). However, in presence of chronic stress situations, the strong induction of HO-1 may result to be cytotoxic due to extreme iron accumulation (27). In exosomes, the increased HO-1 levels reflected the cytosolic situation and could be a part of the non-specific defense against inflammation and ROS increment into psoriatic KCs. In another study iron levels measured through heme-oxygenase activity as other acute phase reactants present in EVs were used to track disease progression and quantify acuteness (28).

The mRNA cargo carried by EVs from IL-17A treated HaCaT cells was enriched by neutrophil and lymphocyte chemoattractant C-X-C motif ligand (CXCL)1, CXCL3, CXCL5, CXCL6 and C-C motif ligand (CCL)20 and AMP Defensin-β2 (hBD2). The aforementioned results were confirmed through neutralization of rIL-17A by using anti-17-A antibody (*i.e.*, Secukinumab). When administered to the treated cells, Secukinumab reversed the CXCL1, CXCL3, CXCL5, CXCL6, CCL20 and hBD2 mRNAs to levels similar to those of the untreated controls. Interestingly, EVs collected from IL-17A treated KCs also induced endogenous expression of Defensin-β2 mRNAs in acceptor cell (16). Furthermore, IL-17A and IL-23 increase the transcription levels of hBD2 and hS100A12 in cells, and hBD2 in EVs. Conversely, IL-17A, TNF-α, IL-22 and IL-23 upregulated the levels of S100 calcium binding protein A12 (S100A12) in the secreted EVs (17).

A massive study of miRNA exosome content from plasma samples of psoriatic patients discovered 246 miRNAs differentially expressed compared to exosomes from plasma of healthy donors. In particular, 166 miRNAs were upregulated whereas 80 were downregulated compared to healthy donors. Among those analyzed, let-7d-3p, miR-125a-5p, -134–5p, -142–3p, -155–5p, -375–3p, -485–5p, -941 and -1228–5p were the most deregulated. Subsequently, a GO enrichment analysis allowed to define the principal biological processes involved in relation to the deregulated miRNAs. KEGG pathway enrichment analysis was directed against miRNAs targets to find the associated pathways. It has been found that cellular metabolic process, cellular process, signal-organism cellular process, metabolic pathways, endocytosis, apoptosis and spliceosome were the most affected among the others (29). The characterization of exosome content of various cell types concurring to the development of the psoriasis, highlights specific miRNA profile for every cell type. Treg derived exosomes are enriched with miR-146a-5p, -150–5p and -21–5p. Th1/Th17 derived exosomes contain high levels of miR-106a-5p, -155–5p and -19a-3p. The possibility to create an *in vitro* model of the psoriasis has been applied to study the miRNA profile from keratinocyte treated derived exosomes: 28 miRNAs are enriched while 114 result downmodulated compared to untreated cells. By evaluating the circulant exosomes into psoriatic patient, it was an interesting highlight as let-7b-5p and miR-30e-5p could be discriminant for the development of cutaneous-only psoriasis with respect to psoriatic arthritis, while miR-1305 dampen could be a master regulator of psoriasis pathophysiology by modulating Wnt pathways (30). Changes in serum miRNA population between patients with plaque psoriasis, psoriatic arthritis and control patients have been found by Lattekivi et al. (31), suggesting that EV mediated communication could be crucial in the pathophysiological development of these diseases. In this study, instead of an overall change in EV bound miRNAs, major shifts in enrichment profiles were discovered. These insights also usefully correlate with other inflammatory diseases such as osteoarthritis (OA). A deregulated miRNA that was previously found to be downregulated in psoriatic skin biopsies, namely hsa-miR-99b-5p, was found to be deregulated in sera collected from patients with plaque psoriasis. This data fits also with the observation that hsa-miR-99b-5p has been positively correlated with keratinocyte hyperproliferation. Hsa-miR-671–3p was found to be down-modulated in patients with arthritic psoriasis compared to the control groups. This miRNA is also deregulated in OA and its role is to regulate the expression of OA correlated genes in chondrocytes and osteoblasts suggesting a possible connection between psoriasis and other inflammatory diseases (32).

Exosome cargo could be defined also by circulating long non-coding RNAs (lncRNAs). Such type of non-coding RNAs (ncRNAs) are characterized by a sequence of more than 200 nucleotides, a secondary structural conformation and regulatory of gene expression function. The lncRNA PRINS (Psoriasis-susceptibility-Related RNA Gene Induced by Stress) can contribute to the pathogenesis of psoriasis by increasing the expression of the anti-apoptotic G1P3 gene but until now the presence of lncRNA PRINS into KCs-derived or immune cells associated to psoriasis-derived exosome is not well understood (33).

Besides nucleic acids and proteins, lipids also have a role in EV mediated communication in normal physiology as in psoriasis. The alteration of EV membranes and cargo in phospholipid composition benefits the uptake in acceptor cells thus facilitating effective cell-to-cell communication. Starting from the observation that plasma lipidic profiles in psoriatic patients are altered, Paolino et al. studied the phospholipid composition of plasma microvesicles and exosomes (34). In psoriatic patients undergoing treatment with Secukinumab, Ustekinumab, Adalimumab, an increased production of microvesicles and exosomes in plasma was recorded, with altered membrane phospholipid composition. Membrane phosphatidylcholine, phosphatidylethanolamine, phosphatidylglycerol and lysophosphatidylcholine were altered in plasma exosomes from psoriatic patients in comparison with those from healthy subjects. Moreover, in plasma microvesicles from psoriatic patients, changes in sphingomyelin and phosphatidylinositol levels were recorded. Interestingly, treatments with the aforementioned drugs seemed to revert the observed lipidic phenotypes. Ustekinumab reverted the phosphatidylethanolamine and phosphatidylcholine levels in exosomes back to levels comparable to those in healthy subjects. Furthermore, a variation in microvesicle and exosome origin was recorded by Takeshita et al. as an increased level of monocyte and endothelial-derived microvesicles in psoriatic patients (32). These data suggest that lipid profiles of the sera of patients could be a potential tool for a quantitative diagnosis and management of psoriasis. A resume about the molecular composition of EVs related to psoriatic disease is shown on **Table 1**.

**Table 1:** Schematic table summarizing the cargo carried by EVs or exosomes, the quality of modulation in the target cell and the resulting effects.

Component	Mediator	Type of regulation	Effects	Origin	References
mRNA	hBD2 CXCL3 CCL20	Upregulated	Pro-inflammatory	HaCaT	(16)
	S100A12 hBD2	Upregulated	Pro-inflammatory	pso-KC	(17)
ncRNA	miR-146a-5p miR-150–5p miR-21–5p	Upregulated	Pro-inflammatory Onco-regulatory	Treg	(30)
	miR-106a-5p miR-155–5p miR-19a-3p	Upregulated	Pro-inflammatory Onco-regulatory	Th17	(30)
	miR-30e-5p let-7b-5p	Downregulated (in PsA)	Discriminates between cutaneous and arthritic psoriasis	Blood	(35) (30)
	miR-1305	Downregulated	Amelioration of psoriasis development	Blood	(30)
	miR-381–3p miR-365–5p miR-4488 miR-619–5p	Upregulated	Role in Th1/Th17 polarization.	pso-KC	(36)
	miR-4505	Upregulated	Induction of M1 macrophage differentiation.	pso-KC	(37)
	ASO-210	Delivery	Anti-inflammatory	MSC-EVs	(38)
	circ_0024028	Expression	Sponge activity for miR-486–3p.	pso-KC	(39)
Protein	OLFM4	Expression	Positively correlated with severity of GPP Pro-inflammatory	pso-Neutrophils	(40)
	JPH203	Expression	LAT-1 inhibitor.	pso-KC	(41)
	Heme-oxygenase sTfR	Upregulation	Anti-inflammatory	Blood	(28)
Lipids	Pristimerin	Expression	Anti-inflammatory	Melanoma cells	(42)
	Phosphatidylcholine Phosphatidylethanolamine Phosphatidylglycerol Lysophosphatidylcholine	Upregulation	Enrichment of EV plasma membrane in psoriasis patients	pso-KC	(34)

RNAs, proteins or lipids that are expressed ex novo in EVs or exosomes and are delivered in innovative therapy strategies have been labeled as “Expression”.

## 3 EV cellular trafficking and microenvironment

Jiang et al. demonstrated the influence of EVs originating from KCs treated with cytokines during psoriatic development, specifically in Th1 and Th17 polarization (36). EVs derived from cytokine-stimulated KCs have been shown to influence T cell response to the point of over proliferation and activation, thus leading to psoriasis. Small RNAs such as miRNAs have a major role in regulating CD4^+^ T cell polarization into Th1 and Th17 subsets. Indeed, the sequencing of RNA extracted from these EVs showed that 28 miRNAs were upregulated while 114 were downmodulated. Among these modulated miRNAs, miR-381–3p expression increased in EVs from cytokine-treated KCs and in CD4^+^ T-cells from psoriatic patients. In the receiving cells, IFN-γ, IL-17A, IL-17F, T-box expressed in T cells (T-bet), and RAR-related orphan receptor gamma (RORγt) transcript levels enhancement and IFN-γ and IL-17A protein levels increase were observed. Since miR-381–3p has been positively correlated with the Psoriasis Area Severity Index (PASI) score, a clinical-quantitative scale used to determine the severity of psoriasis cases, such evidence suggests the important role of EV cargo in psoriasis development and management. During the development of psoriasis, the Th cell-keratinocyte axis has a crucial role. Although the underlying mechanism is not entirely clear, it is understood that psoriatic KCs communicate with CD4^+^ cells and induce Th1 and Th17 polarization. This mechanism may also modulate the hyper immune response that is associated with psoriasis. Jiang et al. determined that miR-381–3p is carried from psoriatic cells to CD4^+^ cells through vesicle trafficking, thus polarizing T helper cells towards the Th1 and Th17 phenotype (36). These findings can be considered both as an interesting development in scientific understanding of psoriatic pathogenesis and as an innovative platform to contrast this disease since these KC-EVs could be implemented in immunomodulatory therapies.

Neutrophils are able to amplify the psoriatic inflammatory deregulation by building the Neutrophils Extracellular Trap (NETs), a structure composed by proteins and DNA that promotes hBD2 expression in KCs and the induction of type 17 T helper cells from peripheral blood mononuclear cells. It has been observed that cytokine-treated keratinocyte exosomes are able to activate NF-κB and p38 pathways on neutrophils leading to the production of IL-6, IL-8, and TNF-α, and so promoting the induction of NETosis. The precise mechanism behind this process has been not defined yet but it seems to be fundamental that the inflammatory message from KC has been conveyed through exosomes. The NET exploits its role when the structure is complete and psoriatic KC-derived exosomes could also be a part of this structure (22). The ability of the EVs derived from KCs stimulated with psoriatic cytokines to induce NETs was investigated also by Capriotti et al. (17). This was carried out by exposing primary neutrophils to supernatant derived from HaCatT cells treated with IFN-γ, TNF-α, IL-17A, IL-22 and IL-23. All cytokines, apart from IFNγ, were able to induce the formation of the neutrophil traps (17). Psoriatic lesions are known to be characterized by a heightened migration and motility of KCs. To further investigate this evidence, HaCat cells were incubated in transwells with EVs from untreated or treated with IL-17A and IFN-γ HaCaT cell. While the cells exposed to the IL-17A treated EVs showed no relevant change in motility compared to the untreated controls, those treated with the IFN-γ derived EVs were able to migrate more than the controls. Behind these differences between IL-17A and IFN-γ, the authors speculate that the hypercellularity seen in psoriatic lesions could be connected to the effect of IL-17A of halting cell migration. Interestingly, IFN-γ has an opposite effect on NETosis, modulating the phenomenon more than IL-17A (17).

The miRNAs activity can be modulated by the action of the circular RNAs that are able to capture miRNAs due to their structure. Circ_0024028 is a circular RNA highly expressed into psoriasis lesions and IL-22 stimulated HaCaT cells. Moreover, it has been demonstrated that in HaCaT cells Circ_0024028 upregulation was associated with cell proliferation and migration and its expression is dependent to IL-22 stimulus in a concentration-dependent manner. With an elegant demonstration Zhang et al. have found that circ_0024028 can be accumulated into exosome in a specific manner and that exosomes are able to spread circ_0024028 into surrounding cells. Probably the action of circ_0024028 is related to the sponge of miR-486–3p that is an inhibitor of retinoblastoma (pRB) and AKT serine/threonine kinase 3 (AKT3) genes and an activator of extracellular matrix protein 1 (ECM1) level (39).

The ability of neutrophil-derived vesicles to interact with immune cells for the orchestration of the adaptive immune response is well documented. Shao et al. determined that, Olfactomedin 4 protein (OLFM4), was expressed in exosomes from neutrophils collected from generalized pustular psoriasis (GPP) (40). This protein belongs to the olfactomedin family and is known to be an anti-apoptotic and tumor promoting factor. In their work, the authors approached exosome characterization from a proteomic standpoint analyzing EV cargo from healthy and GPP subjects. OLFM4 was not only found in mRNA form in psoriatic neutrophils but also as a protein in circulating exosomes (determined through Western Blot), shedding interesting information on cell-to-cell communication between neutrophils and psoriatic KCs. Interestingly, when recombinant OLFM4 was introduced in KCs, a spike in CXCL1, CXCL2, CXCL8, and CCL20-containing exosomes was registered. This expression phenotype is typically associated with a psoriatic microenvironment, which leads to increased proliferation and migration of neutrophils and other immune cells to the inflamed area. Thus, the role of OLFM4 has been correlated with the exosome-assisted pathogenesis of GPP.

Mast cells derived exosomes contain phosphatidylcholine 2-acylhydrolase (PLA2) and are able to bind CD1a on T lymphocytes, thus stimulating an inflammatory response. This inflammatory response was determined by Enzyme-Linked immuno-SPOT (ELISPOT) experiments on T cells from healthy and psoriatic subjects stimulated with mast cells derived exosomes. T cells from psoriasis patients had a greater IFN-γ, IL-17 and IL-22 production due to an increased CD1a response compared to healthy individuals. Such CD1a high T cells were preferentially localized near the lesional skin but could also be found into the non-lesional skin and peripheral blood. PLA2 was produced by endogenous cytosolic phospholipase A2 group IV D (PLA2G4D) that was expressed in mast cells and KCs within psoriatic lesions, loaded into exosomes and transferred to CD1a-expressing target cells in a clathrin-dependent manner (43).

Psoriasis is characterized by the increase of the asymmetric division of the basal stem cells. Such situation is caused by the hyperactivation of the Par3/mInsc/LGN signaling pathway. Moreover, proteinase-activated receptor (PAR) proteins cooperate with atypical protein kinase C (aPKC) λ to induce skin tumor and to modulate inflammatory signaling. It has been observed that, during psoriasis, macrophages show high levels of Par3 expression. Exosomes derived from psoriatic macrophage containing Par3 are able to induce asymmetric division of the basal stem cells and inflammation when inoculated in mice skin (44).

**Figure 1:**
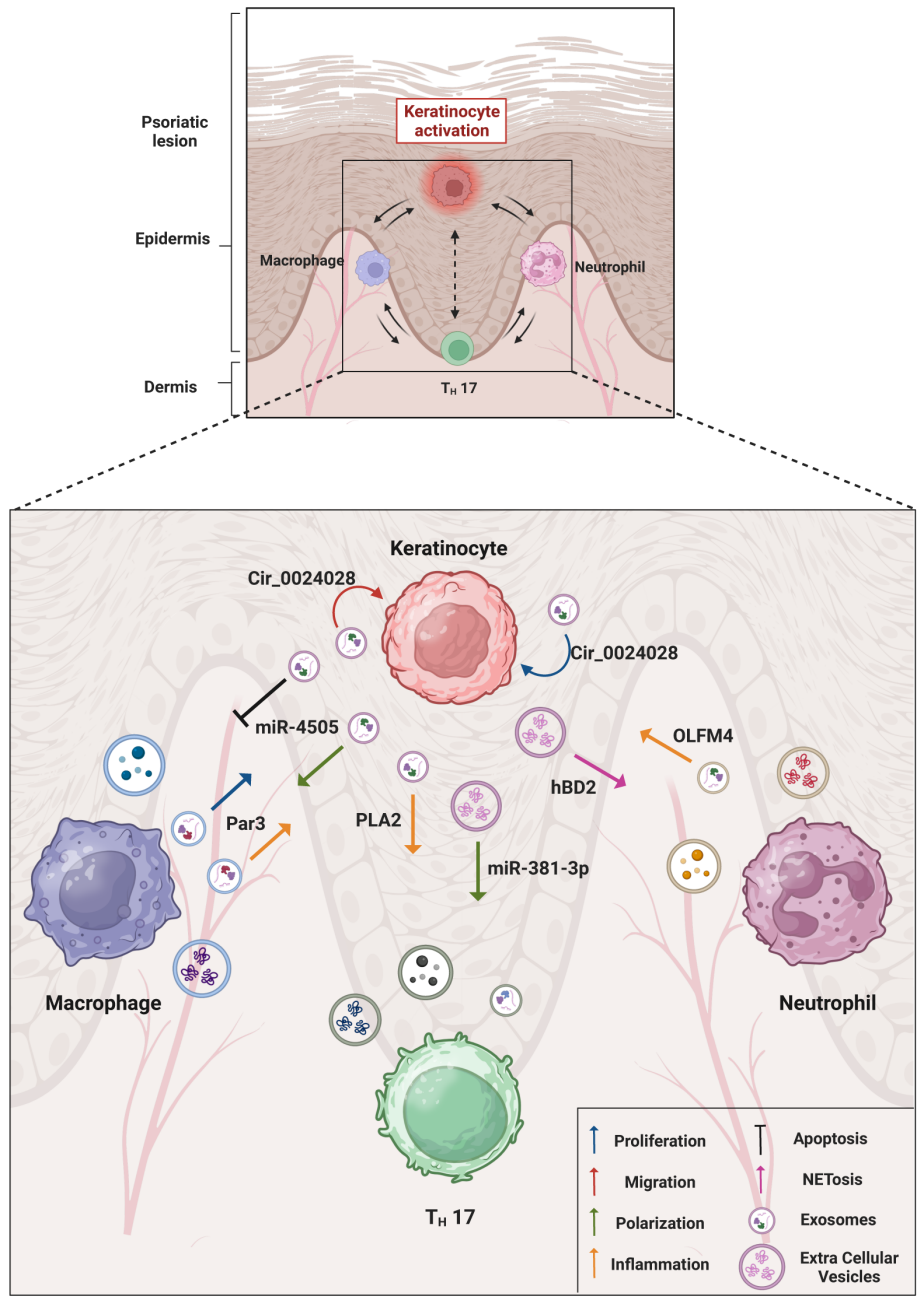
Overview of keratinocytes and immune cells interactions in psoriatic microenvironment. The cartoon summarizes the main molecular mediators found within the EVs and elucidates the resulting inflammatory effects obtained through EV trafficking. An emphasis has been given on the cell-to-cell communication between psoriatic keratinocytes and immune cells (macrophages, T helper lymphocytes and neutrophils) localized in the lesion area. The role of EVs in the psoriatic disease, in particular in the composition of psoriasis-associated secretome and microenvironment indicates the EV involvement in the spreading of disease mediators and in the possible associated comorbidities. Created with BioRender.com.

Psoriatic KCs show a low expression of the vitamin D receptor (VDR). It has been demonstrated that it is possible to induce M1 polarization and inhibition of apoptosis by stimulating macrophages with HaCaT cells derived-VDR deficient exosomes. Moreover, starting from the knowledge that miR-4505 was highly express in psoriatic skin it has been observed that VDR deficient HaCaT cells showed miR-4505 overexpression and the exosomes produced by these cells exerted their M1 polarization and anti-apoptotic activity through the delivery of miR-4505. The M1 polarization of the macrophages, then, seems central in maintaining the inflammation (37). A table resuming all the interaction mediated by EVs between KCs and other immune cells located into psoriatic microenvironment has been represented in **Figure 1**.

## 4 EVs and therapeutic applications

Zhang et al. demonstrated that EVs derived from IFN-γ treated mesenchymal stem cells (MSC-EVs) have the ability to effectively modulate the proliferation of peripheral monocellular cells and T cells in a psoriatic setting (38). MSC-EVs decreased the intensity and presence of hallmark psoriatic symptoms such as skin thickness, scaling and erythema, but also decreased the production of pro-inflammatory cytokines such as IL-6, IL-17A, IFN-γ and TNF-α. Interestingly, Th cell subsets were also modulated with less exhausted Th17 cells and more Th2 cells. Furthermore, these types of cells were shown to be potentially effective tools in disease management. Indeed, antisense nucleotides, in this case ASO-210, were delivered with more efficacy by using MSC-EVs. Nucleotide stability was also heightened, resulting in a successful control of immune imbalance which is at the core of psoriatic development. The ability of EVs to efficiently carry and protect bioactive cargo has been exploited in experiments using innovative compounds. In one case Jiang et al. used ultraviolet induced EVs from cultured KCs to transport an inhibitor of LAT1 (JPH203) with the purpose of blocking the mTOR pathway through leucine sequestration (41). Moreover, the exposure of KCs to UVB radiation caused an accumulation of IL-1 receptor antagonists that, when released in an *in vitro* model, effectively decreased IL-1 mediated inflammation. Positive results of the *in vivo* treatment were also the blocking of NF-κB pathway. In successive *in vivo* psoriasis imiquimod (IMQ)-induced murine experiments, the EVs significantly reduced the typical psoriatic symptoms like acanthosis as well as suppressing the over-active immune reaction, pointed out by the IL-17 release and Th17 expansion (41).

MSCs-exosomes are known to dampen chronic inflammation associated with bowel disease, atopic dermatitis and chronic graft versus host disease (GVHD). On the other hand, EVs derived from umbilical cord blood mononuclear cell are able to downmodulate the expression of inflammatory mediators namely IL-6, IL-8, CXCL10, Cyclooxygenase 2 (COX2), S100A7, and hBD4 in a 3D model of psoriatic skin. When the umbilical cord blood mononuclear cell derived EVs had been administered to IMQ-treated mice the inflammatory dampening was incomplete suggesting a use in combination with standard therapies (45). MSC derived exosomes are able to reduce the expression of IL-17, IL-23 and C5b-9 in IMQ treated mouse skin. Zhang et al. have been tested for topical application demonstrating that they remain along the stratum corneum. Here they can regulate complements components, one of the most induced molecules by NETosis. Since neutrophils are the major producer of IL-17 during psoriasis it could be reasonable to hypothesize that the MSC exosome activity into the *stratum corneum* can finally damp the inflammatory mediators into epidermis (46). A study confirmed the ability of umbilical cord blood mononuclear cell derived exosome to reduce inflammation. Specifically, they reduced the expression of CCL20, IL-17 and IL-23 in IMQ-induced mice and treated HaCaT cells. The entire inflammatory process of psoriasis needs the orchestration of various factors. Mature DCs participate to the development of the disease through the continue production of IL-23 that activating Th17 cells, finally, producing an abnormal production of IL-17, IL-21 and IL-22 and KCs deregulation. The use of umbilical cord blood mononuclear cell derived exosome can reduce the secretion of IL-23 by DCs. Moreover, the phosphorylation of STAT3 is a central mechanism of the IL-17/-23 axis and hucMSCs-Exo were able to inhibit this process in the epidermis of IMQ-induced psoriatic mice and in HaCaT cells (47).

The therapeutical uses of exosomes derived and modified from tumor cells, immune cells or mesenchymal stem cells could be promising. A characteristic of tumoral exosomes, as is the case of melanoma derived exosomes, is the high presence of programmed death-ligand 1 (PD-L1) expressed to achieve immune escape. Jia et al. engineered exosome derived from melanoma cells by introducing a natural anti-inflammatory triterpenoid substance called Pristimerin. The combinatory activity of PD-1/PD-L1 interaction and Pristimerin allowed the engineered melanoma-derived exosomes to dampen inflammation when administrated to psoriatic skin more than the sum of each treatment. The activity of the immune infiltrate in the psoriatic skin is fundamental for the development of the disease. Macrophages are one of the most important immune cells that collaborate into psoriatic inflammation promotion. Engineered exosomes derived from melanoma cells are able to reduce the inflammatory macrophages infiltrate and to drive their polarization into M2 subset. In addition, the interaction between PD-L1 on the surface of the exosomes and PD-1 on the surface of immunosuppressive T cells produced the exhaustion of this kind of T cell so improving the inflammatory status of the affected skin (42).

Correlation between psoriasis and other inflammatory diseases and microbiota diversity has been a subject of scientific interest as of recent years. In order to study microbial skin heterogeneity, α and β diversity is usually employed. The former represents the diversity within a sample of an ecological community, while the latter is used to measure how much two distinct communities differ. In this setting, interesting developments have also arisen in the relationship between host and commensal bacteria of the skin, intestinal mucosa and other tissues. Indeed, communication between the host and non-pathogenic bacteria are crucial for certain mechanisms such as tissue healthiness, functionality and defense against pathogens and can be carried out through EVs. Chang et al. expanded on this subject by performing metanalyses of skin and intestine microbiota by analyzing the origin and diversity of serum EVs (48). By searching EV contents for Microbial Associated Molecular patterns (MicroAMPS), their findings pointed out a lower richness and microbial diversity in intestinal and skin microbiota in psoriatic patients. This is in line with previous studies in which a decreased alpha-diversity was measured in the intestinal microbiota of psoriasis patients, thus speculating an interesting correlation between dysbiosis, psoriasis and Inflammatory Bowel Disease (IBS) related diseases. Furthermore, the authors found that the presence of *Staphylococcaceae*, most importantly *S. aureus*, took up a higher percentage of the entire microbiota in psoriatic patients compared to healthy controls. This Gram-positive bacterium is responsible for the inflammation of skin mucosa and KCs through allergic reaction and release of alpha-toxins. *S. aureus* infection has also been highly correlated with psoriasis severity (49). In conclusion, analyzing EV content for Metabolism Associated Molecular Patterns (MAMPS) or deviation from biochemical homeostasis could represent a viable option for psoriasis diagnosis and management. Commensal bacteria play an active role in skin health by promoting regeneration after injury and by impeding pathogen colonization. Among the many species that inhabit the skin microbiota, *S. epidermidis* is one of the most active in protection against pathogens and immunomodulation during inflammation. Chang et al. reported that in psoriatic skin *S. epidermidis* and *Propionibacterium acnes* were under-represented, suggesting a possible role of these species in psoriasis contrast. Since the mechanism through which the immunomodulation occurs is unknown, it is possible to hypothesize that EVs could play a role in signal transduction (48). Gomez-Chavez et al. presented a study in which EVs extracted from two *S. epidermidis* strains, one commensal (ATC12228) and a second one of clinical origin (983), were used to test the effect on a psoriatic skin platform, namely the *in vivo* IMQ-induced murine model (50). In an *in vitro* experiment with the keratinocyte cell line HaCaT, both the ATC12228 and the 983 derived EVs were capable of inducing proinflammatory IL-6 expression, although EVs from the clinical strain induced a higher level of other inflammatory mediators like Vascular endothelial growth factor-A (VEGF-A), LL-37, IL-8, and IL-17F. In the *in vivo* experiment using the IMQ-induced murine model the ATC12228 EVs actually reversed typical psoriatic symptoms like acanthosis and cell infiltration as well as VEGF-A, IL-6, IL-23, IL-17F and IL-36 mRNA transcription. Conversely FoxP3 expression had no significant change in expression and IL-36 receptor antagonist was found to be increased. With these findings the authors evaluated the therapeutic potential of *S. epidermidis* EVs in regulating the immune response in psoriatic skin (51).

## 5 Discussion

This is a state of the art update on the importance and future perspectives of EVs, the emergent regulatory biological structures in virology, immunology and pathology, as explored by researchers from both inside and outside the EV community. This work focused on the role of EVs in the pathogenesis of the chronic inflammatory disease, psoriasis.

EVs are heterogeneous, membrane-enclosed nanostructures that are evolutionally conserved and released by cells of living organisms. EVs are identified as an alternative secretory mechanism for cytokines/chemokines and the regulatory role of specific cytokines in vesicle release, trafficking and/or content is almost recognized. Their role as important mediators of cell-to-cell communication in physiologic and pathologic conditions has emerged in the last two decades. The composition of the EV cargo is diverse and critical for intercellular communication. EV cargo is defined by the lineage of the parental cells and their state of activation. EVs protect their cargo, *e.g.* miRNAs secreted into the microenvironment are preserved from serum RNAse degradation as a result of being encapsulated inside a double membrane structure; once transferred to recipient cells, EVs could promote inflammation by regulating gene expression leading to multiple physiological changes in cell proliferation, migration, intercellular communication and/or stromal modification. The inflammatory microenvironment is characterized by the presence and activity of specific combinations of molecular, cellular and sub-cellular mediators derived from both immune and non-immune cells that collectively contribute to inflammation. Among the sub-cellular mediators are EVs that are derived through budding processes from cellular membranes and are secreted into the extracellular space by many cell types. Many inflammatory-associated pathological disorders, ranging from autoimmune diseases to cancer (52–55), are mainly characterized by a microenvironment with specific inflammatory elements (*i.e.*, immune cells infiltrate, cytokines, chemokines, AMPs and Damage-associated molecular patterns). The role of EVs in the psoriatic disease, in particular in the composition of psoriasis-associated secretome and microenvironment indicates the EV involvement in the spreading of disease mediators and in the possible associated comorbidities.

However, the studies are still at their infancy in the dermatological field. The sections on different topics of recent EV studies, from EV specificity, production, cargo and extracellular functions, as well as some pilot therapeutic applications, aim to address the emerging challenges up to date limiting the broader translational use of EVs. The molecular cargo and the origin of EVs related to psoriatic disease is outlined in **Table 1**. The main interactions mediated by EVs between KCs and other immune cells located in the psoriatic microenvironment are represented in **Figure 1**.

Likewise, the highlighted new strategies and more comprehensive studies appear to be in progress to identify EV subpopulations with high accuracy and selectivity. Notably, new technologies have flourished in recent years allowing future applications to benefit from EVs’ identification and profiling, with the aim to detect and treat inflammatory skin diseases.

## Author contributions

MI: Conceptualization, Writing – original draft, Writing – review & editing. LG: Writing – original draft, Writing – review & editing. PR: Writing – original draft, Writing – review & editing. SS: Writing – original draft, Writing – review & editing. NB: Supervision, Writing – review & editing. IP: Supervision, Writing – review & editing. ET: Supervision, Writing – review & editing. NS: Supervision, Writing – review & editing. CP: Supervision, Writing – review & editing. GM: Conceptualization, Funding acquisition, Supervision, Writing – original draft, Writing – review & editing. GR: Conceptualization, Funding acquisition, Supervision, Writing – original draft, Writing – review & editing.
